# Development and evaluation of three-dimensional transfers to depict skin conditions in simulation-based education

**DOI:** 10.3205/zma001669

**Published:** 2024-04-15

**Authors:** Kai Philipp Schnabel, Andrea Carolin Lörwald, Helmut Beltraminelli, Miria Germano, Beate Gabriele Brem, Sandra Wüst, Daniel Bauer

**Affiliations:** 1 University of Bern, Faculty of Medicine, Institute for Medical Education, Bern, Switzerland; 2 Inselspital, Bern University Hospital, University of Bern, Department of Dermatology, Bern, Switzerland; 3 Università della Svizzera Italiana (USI), Department of Dermatology, Ente Ospedaliero Cantonale (EOC), Bellinzona, Switzerland; 4 University of Bern, Faculty of Medicine, Institute of Primary Health Care, Bern, Switzerland

**Keywords:** transfer tattoo, medical moulage, health professions education, summative assessment, OSCE

## Abstract

Modern medical moulages are becoming increasingly important in simulation-based health professions education. Their lifelikeness is important so that simulation engagement is not disrupted while their standardization is crucial in high-stakes exams. This report describes in detail how three-dimensional transfers are developed and produced so that educators will be able to develop their own. In addition, evaluation findings and lessons learnt from deploying transfers in summative assessments are shared.

Step-by-step instructions are given for the creation and application of transfers, including materials and photographic visualizations. We also examined feedback on 10 exam stations (out of a total of 81) with self-developed three-dimensional transfers and complement this with additional lessons learnt.

By the time of submission, the authors successfully developed and deployed over 40 different three-dimensional transfers representing different clinical findings in high-stakes exams using the techniques explained in this article or variations thereof. Feedback from students and examiners after completing the OSCE is predominantly positive, with lifelikeness being the quality most often commented upon. Caveats derived from feedback and own experiences are included.

The step-by-step approach reported can be adapted and replicated by healthcare educators to build their own three-dimensional transfers. This should widen the scope and the lifelikeness of their simulations. At the same time we propose that this level of lifelikeness should be expected by learners as not to disrupt simulation engagement. Our evaluation of their use in high-stakes assessments suggests they are both useful and accepted.

## Abstract (de)

Moderne medizinische Moulagen gewinnen in der simulationsbasierten Ausbildung der Gesundheitsberufe zunehmend an Bedeutung. Ihre Authentizität unterstützt den reibungslosen Ablauf der Simulationen, während ihre Standardisierung bei bestehensrelevanten Prüfungen von entscheidender Bedeutung ist. Dieser Bericht bietet eine detaillierte Beschreibung zur Entwicklung und Produktion dreidimensionaler Transfer-Tattoos, um Lehrpersonal in die Lage zu versetzen, eigene Transfer-Tattoos zu entwickeln. Darüber hinaus werden Evaluationsergebnisse und Erkenntnisse aus dem Einsatz von Transfer-Tattoos (Moulagen) in Prüfungen dargestellt.

Schritt-für-Schritt-Anleitungen für die Erstellung und Anwendung von Transfer-Tattoos werden präsentiert, einschließlich Materialien und fotografischer Visualisierungen. Zusätzlich haben wir das Feedback von 10 Prüfungsstationen (von insgesamt 81) mit selbst entwickelten dreidimensionalen Transfer-Tattoos untersucht und ergänzen dies mit Erfahrungen aus der praktischen Anwendung.

Zum Zeitpunkt der Einreichung haben die Autor*innen erfolgreich über 40 verschiedene dreidimensionale Transfer-Tattoos entwickelt und eingesetzt, die unterschiedliche klinische Befunde in bestehensrelevanten Prüfungen darstellen, wobei die in diesem Artikel erläuterten Techniken – oder Variationen davon – verwendet wurden. Das Feedback der Studierenden und Prüfer*innen nach Abschluss der OSCE war überwiegend positiv, wobei die Authentizität (Realitätsnähe) als häufigste kommentierte Qualität hervorgehoben wurde. Vorbehalte, die sich aus den Rückmeldungen und unseren eigenen Erfahrungen ergeben, werden hier ebenfalls aufgeführt.

Der beschriebene Schritt-für-Schritt-Ansatz kann von Lehrpersonen im Gesundheitswesen angepasst und repliziert werden, um ihre eigenen dreidimensionalen Transfer-Tattoos zu erstellen. Dies sollte helfen, den Umfang und die Realitätsnähe ihrer Simulationen zu erhöhen. Gleichzeitig verweisen wir darauf, dass den Studierenden dieses hohe Maß an Authentizität auch vertraut sein sollte, um ihr Engagement in der Simulation nicht zu beeinträchtigen. Die Ergebnisse unserer Auswertungen deuten darauf hin, dass der Einsatz der Moulagen in bestehensrelevanten Prüfungssituationen sich als nützlich erwiesen hat und breit akzeptiert war.

## 1. Introduction

Modern medical moulages are widely expanding the range of clinical simulations that allow simulated patients (SPs) to display visible findings on their skin, which they would otherwise not be able to physically portray by themselves no matter how experienced (e.g. simulating a rash). Moulages allow presentation of a myriad of traumatological [1], [2], dermatological [3], [4], and medical [5] findings. They involve methods such as temporary tattoos [6], [7], prosthetics [4], [8], and special effects makeup [9]. They are used in teaching [10], [11] and assessment [12], including high stakes standardized assessment [8] throughout health professions at all educational levels [13]. 

The role of moulages can be pivotal for learner engagement and the course of simulation in general. Whereas *lifelike *moulages add to the authenticity of a simulation’s similarity in relation to clinical practice, *insufficiently lifelike* moulages could constitute a dissimilarity from reality. Such disparities might require learners to mentally disregard them as irrelevant, possibly disrupting engagement and breaching the simulation [14], [15]. Ensuring that moulages are sufficiently lifelike is therefore essential. Deliberations on how to determine the right level of lifelikeness and how to monitor the quality of moulage development and deployment are presented in detail in our paper on two-dimensional transfer tattoos [13]. That paper also gives an oversight into different contemporary moulage techniques and their respective pros and cons in simulation [13].

Although moulages are already widely used in healthcare education worldwide, descriptions of their development and properties are often not elaborate enough to allow for easy reproduction. The aim of this article is to enable educators with some prior experience in moulage techniques to produce their own three-dimensional moulages, aiming for a level of quality that allows the moulages to be used in summative assessments. In this report we describe in detail the process from the development of a prototype to the production of three-dimensional flat mould transfer moulages ready for application. These transfers are especially useful for deployment in summative assessments, as they can be prepared well in advance to the exam, are quickly applied onto the SP on the day of examination and offer high levels of standardization and lifelikeness. Their thin edge makes the transition between the moulage and the SP’s own skin very unobtrusive. Their main disadvantages are that they are single-use and relatively resource intensive to develop. This article provides guidance on how to effectively develop three-dimensional transfers and includes evaluation data from three high-stakes Objective Structured Clinical Examinations (OSCE) utilizing said transfers to depict hyper-realistic skin lesions. 

## 2. Project description

The methods section first describes the development process for three-dimensional transfers, then outlines the evaluation approach taken. We have added additional visualizations of the development process as supplementary material (see attachment 1 ).

### 2.1. Preparation

To facilitate the successful development of three-dimensional transfers, a team was pulled together which combined medical and simulation expertise as well as make-up artistry. Together, clear criteria for assessing the lifelikeness of each transfer and ensuring that it meets the educational needs were established. For example, it was determined that wound moulages for mass casualty simulations that will only be glimpsed at for the purposes of triage have different requirements than dermatologic moulages in practical summative assessments which would be scrutinized intensively by examinees in order to develop their diagnosis. Criteria to be considered before modelling started included the following:

1. the finding itself (location on the body, number of lesions and their arrangement, efflorescences, size (width, length, diameter; height/depth), shapes, margins, colour ranges and homogeneity, textures, consistency, mobility against surrounding structures, secretions (incl. blood, pus), toppings (flaking, keratosis, crusts), possibly odours (burn/smoke, pus); and
2. the surrounding SP skin (colour, age, conditions such as scars, hairiness, nails, bandages).

It also had to be determined who the intended wearer of the moulage was going to be (an SP or a simulator/model). This list of criteria is not exhaustive. The following describes the development of a three-dimensional transfer with a flat base that can be mounted on flat surfaces, like a cheek, back or thigh. 

### 2.2. Preparing a first model (positive)

Using representative, high-resolution photographs of the specific skin condition and theoretical knowledge of the condition’s pathophysiology, the makeup artist prepared a three-dimensional copy of the affliction out of non-hardening modelling dough, mounted on a flat plastic board, using regular metal modelling tools. Once the copy was finished, two walls of modelling dough were made surrounding it, the inner slightly higher than the model, the outer slightly higher than the inner wall yet. The central model constitutes a positive copy of the original condition. 

### 2.3. Preparing a silicone cast flat mould (negative)

While wearing gloves, platsil (silicone) components were weighed according to both the manufacturer’s specifications and the size of the cast mould. Components A and B were blended in a cup and stirred with a spatula. Once mixed, the hardening component was added and stirred in, after which the Hi-Ro-Slip release agent was measured according to manufacturer’s specifications, then added and stirred in. The silicone was then poured onto the positive modelling dough model, including the intramural compartment and left to harden for around 2 hours (depending on the size of the mould and the silicone’s consistency). Once hardened, the silicone cast mould was lifted from the board, resulting in a negative copy of the original condition. The silicone mould was then stored in a freezer at -18°C for 4-6 hours (cf. Hi-Ro-Slip specifications for the exact time). The board with the modelling dough positive was preserved for later use so that in case of mass production additional silicone moulds could be prepared from the initial positive copy).

### 2.4. Preparing the transfer

The Transfer Tattoo paste (having chosen the right colour tone) was filled into the silicone mould. This paste is commercially available in different colour tones, but it can also be mixed on site e.g. by adding acrylic paint to transparent paste. For darker skin tones, using transparent paste only may work just as well as approximating the SP’s skin tone. From a sheet of transparent Transfer Tattoo release film, a section slightly bigger than the whole paste was cut and then placed onto the paste. By running a spatula over the release film, the paste was slightly pressed into the mould and superfluous paste pushed sideways into the surrounding cavity. The whole mould was then placed in a freezer at -18°C until the paste had hardened. The length of time again depended on the size of the mould but took approx. 2 hrs. Once hardened and removed from the freezer, the Transfer Tattoo release film was immediately and carefully removed from the silicone mould with the hardened paste now affixed to the release film, detaching it from the silicone mould. If the hardened paste was stuck to the mould instead of the release film, this step was repeated, now lining the mould with Probondo Release & Seal or petroleum jelly before filling it with the Tattoo Paste. The ring of superfluous paste surrounding the finding could then be pried off manually. What remained constituted a somewhat sticky positive of the finding which was left to dry for several days (or depending on the size of the transfer) in a protected, draft and dust-free environment. The silicone mould was preserved for later use.

### 2.5. Colouring and finishing the transfer

Once dried, the transfer was coloured using the initial documentation and photographs as a visual reference. This was done with 96% isopropyl soluble colours. Colours were prepared on a palette and once the right hues were found, painted directly onto the transfer. Since the transfer was transparent with a light fleshy colour, only the pathology had to be painted on, excluding healthy skin. Once colouring was finished and the colours had dried, the transfer was ready to be fixed onto the Transfer Tattoo paper. From a sheet of Transfer Tattoo paper, a section corresponding to the size and shape of the finding was cut. Taking the Transfer Tattoo release film, the transfer was placed on the correct side of the paper, pressing it well to fix the transfer onto the paper without tearing it. Once lifelikeness of the moulage was confirmed (see next step), this form of the transfer can be stored in a cool place for later use (usually several weeks, up until 3-6 months).

### 2.6. Application

Lifelikeness was determined by fixing a trial transfer onto the manikin or SP by removing the release film carefully and placing the transfer onto the intended surface, paper-side up. The paper was then soaked well in water, carefully removed, waiting for the remaining water to evaporate. A cotton swab was soaked with 96% isopropyl alcohol and any residual paste on the edges was removed. A fresh swab was used to cover the whole transfer, going slightly beyond the edges with a sealer for fixation, increasing robustness and preventing stickiness. Once completely dry, additional effects could be added (coagulated blood, desquamation, pus, gloss). The moulage’s effective lifelikeness was then checked [14]. If deemed insufficient, earlier stages were revisited, e.g. by adapting the original first modelling dough positive if the shape or size were not satisfactory, or possibly just by re-colouring. Once adequate, production was initiated. 

### 2.7. Removing the transfer

To remove the transfer, cotton pads were soaked with makeup remover and used to carefully soak and rub on the transfer’s edges. Once removed, additional skin care products were applied as necessary. Transfers were always removed once the teaching or exam session had finished.

### 2.8. Material

The approach described for the production of three-dimensional transfers is generic and should work with materials from other producers. We state the materials used in our workshop for reproducibility (see table 1 (Tab. 1)).

Additional material used: gloves, cups, digital scale, wooden spatula, paintbrushes, 96% isopropyl, modelling tools, plastic board, colour fan deck, cotton swabs, cotton pads. Relevant safety standards were maintained throughout production.

### 2.9. Evaluation

To evaluate the use of three-dimensional transfers in assessments, we analysed the candidates’ and examiners’ feedback from three end-of-term high-stakes 5^th^ year OSCEs (2019-2021), delivered over three days each, and each with 9 different stations per day (cf. table 2 (Tab. 2)). Both parties were asked to provide anonymous feedback at the end of each exam day, both general and regarding the specific stations via the faculty’s evaluation platform. In order not to cue the respondents’ feedback, no specific items regarding the transfers were included. Given the context of the evaluation right after the exam day, we assumed potential criticism of transfers would surely be reported. In this evaluation, only feedback regarding exam stations utilizing three-dimensional transfers was included. We thus dismissed evaluation data on stations using no or other moulage types such as makeup effects, two-dimensional transfers or other visualizations of pathologies, cf. table 2 (Tab. 2).

Feedback from candidates and examiners was categorized (DB, ACL) as to referring either to the transfer directly or the presentation of the clinical finding of said case. Any comment on a transfer was further categorized in terms of whether it constituted praise or a suggestion regarding the moulage’s quality or its use in the scenario. 

## 3. Results

**Figure 1:**
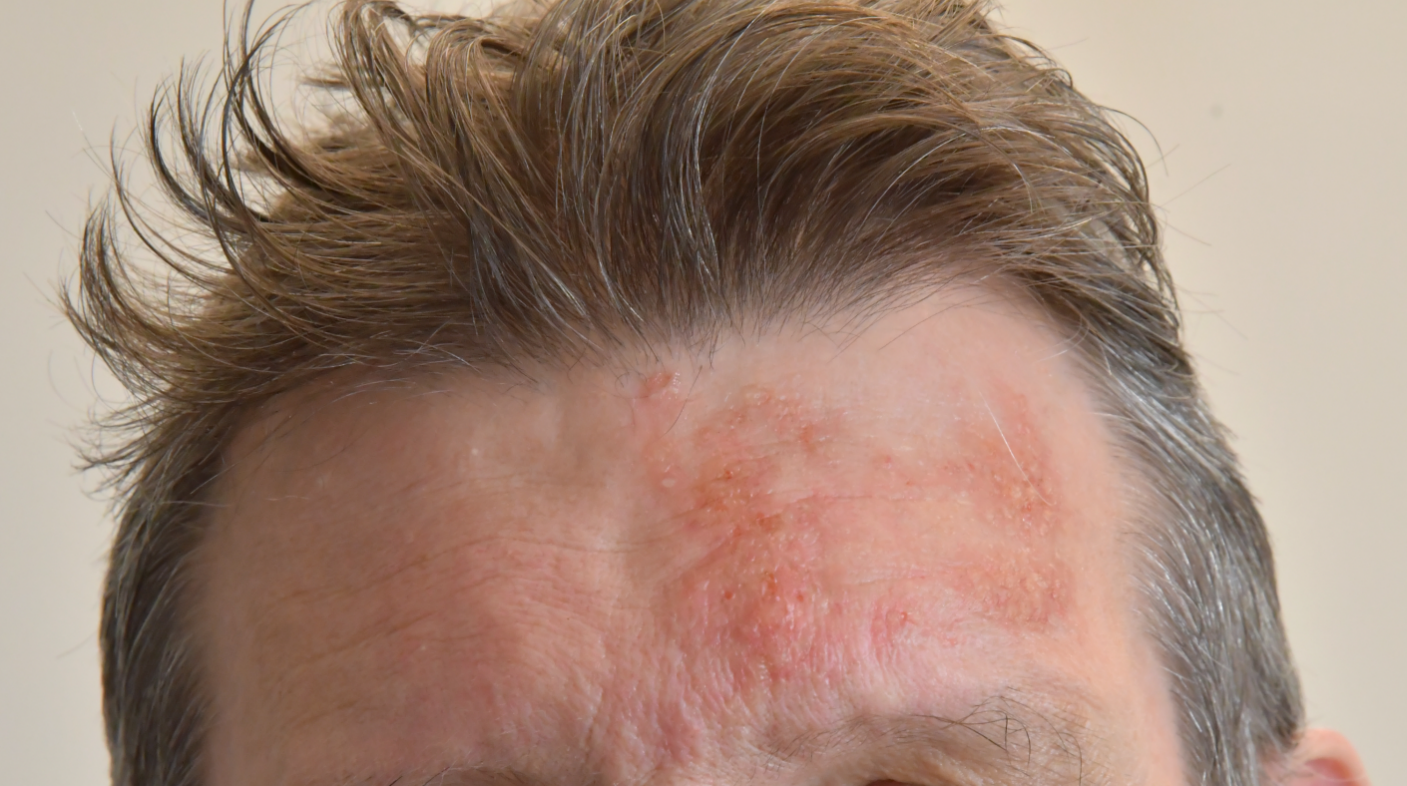
A three-dimensional transfer depicting herpes zoster on an SP’s forehead. This particular transfer was produced in two layers, using clear Probondo paste to allow for clear vesicles and a second layer in a light flesh colour to mimic skin.

Using this technique, we were able to produce about 40 different three-dimensional transfers, some crucial for reaching a diagnose and producing an adequate management plan like acne, burns, cuts, scabies, atopic eczema, psoriasis and herpes zoster (cf. figure 1 (Fig. 1)), and others of a more supportive character (e.g., scars), used in both simulation-based assessment [16] and clinical skills teaching. 

The number of (re-)visits with the expert for further feedback and subsequent re-editing depended primarily on the details of the affliction in question and the resulting technical complexity of the transfers. While the development of something less specific as scars took little time and little expert feedback, development of a second-degree burn took considerably longer as the blisters had to be created by adding encapsulating gelatine bubbles into the transfer to guarantee the correct haptic experience. Development of a three-dimensional transfer of average complexity from scratch took the makeup artist between eight to ten working hours in total. 

Scrutiny of feedback over three years of OSCE evaluations revealed that 16 of 136 candidates’ comments (12%) and 4 out of 45 examiner comments (9%) touched on the transfers, referring only to specific feedback to stations utilizing three-dimensional transfers. 

There were both positive and negative comments regarding the lifelikeness of the transfers and several comments regarding other aspects of the transfers. Of the 16 mentions by candidates, 11 mentioned the transfers positively and 5 voiced some kind of criticism. 7 comments did not refer to the transfers lifelikeness but other aspects instead. Of the 4 comments by examiners, 2 were positive nature while 2 criticized the visualizations in some form. 4 comments touched upon other aspects of the transfer deployment. Due to the low number of mentions of the transfers overall, we do not report on the results quantitatively but narratively in the following.

Some candidates praised the transfers’ lifelikeness directly, stating, e.g., *“…this was portrayed very nicely by the makeup artist”* or *“very real looking skin lesions”.* (authors’ translations. Concrete pathologies redacted as authors cannot disclose exam cases’ diagnoses publicly). Examiners positively commented on the visual lifelikeness, e.g., *“the [lesion] as entry port for germs was well made, easily recognizable and essential for the pathogenesis”*.

Criticism by the candidates was quite succinct, e.g., *“the injury wasn’t easily visible”* (authors cannot publicly disclose if this was intended ) or *“the dermatological features weren’t represented well”*. Two examiners gave feedback on how the lifelikeness could be further improved, e.g., suggesting that *“the delineation should be sharper”*.

Other comments on the transfers by the examiners were regarding the deployment of the moulages on a didactic level, e.g., *“…an ideal case for using moulages”* or sometimes provided technical feedback, e.g., *“do not place the moulage in a skin fold, otherwise it will stick together”*.

One student reported being irritated by the fact that in addition to the moulage, complementary photographic material was provided and that they found it *“…somewhat confusing that the patient had [the lesions] on the head but that my interpretations still had to be based on the photograph presented”*, while others referred to questions of standardization, e.g., *“it seems that the SPs’ sleeves had slipped back in some cases, spontaneously revealing [the lesions], but unfortunately that did not happen in my encounter”*. Several students also indicated having trouble distinguishing between simulation and reality, unsure if a moulage was in fact a real finding on the SP, or a simulated finding for the purpose of the exam: *“it was a bit confusing that the actress [i.e., the SP] actually had severe [lesions] on her forehead, and it wasn’t entirely clear to me if that was part of the scenario or just her face”*.

Two additional indicators of lifelikeness are worth mentioning, i.e. that none of the examiners vetoed the use of transfers (to which they were entitled, using photographs as a fallback option instead) and post-exam analyses did not indicate any moulage-related checklist items which had to be eliminated. 

## 4. Discussion

The use of moulages has widened the scope of pathologies that can presented in simulations. Although moulages are already widely used, descriptions of their development and properties are often too vague to allow for easy reproduction. With this article we wish to enable educators to produce their own three-dimensional moulages and to avoid the most common beginners’ mistakes in developing these. 

Students’ and examiners’ feedback from three OSCEs including moulage scenarios were scrutinized for comments on the lifelikeness of three-dimensional transfers. From a total of 136 comments on ten moulage cases, 16 comments were identified which referred to the lifelikeness of the moulages. An additional 45 examiner comments on these ten moulage scenarios were analysed, revealing 4 references to the lifelikeness of the moulages. The candidates’ comments regarding the quality of the moulages were predominantly positive., This is rewarding, as this feedback constitutes the basis for any appeal, which is why post-exam feedback is usually replete with criticism. The overall low number of comments additionally suggests that the vast majority of candidates and examiners accept the use of moulage and appreciate their lifelikeness. 

The additional comments beyond their lifelikeness raised one very noteworthy issue regarding the placement of moulages in simulations in general, with several candidates indicating problems in distinguishing between reality and simulation. It seems that in an assessment context, some students will try and differentiate between an SP’s features relevant to the scenario and those supposedly irrelevant [17]. The moulages used appeared to puzzle some in making this distinction, perhaps by being unexpectedly lifelike so they were unable to decide if they should take this finding into account or not. This was quite unexpected. We assume most students will probably accept as an axiomatic fact that they will not be confronted with severely ill SPs in exams and thus accept most of what they see as part of the simulation. On top of that, students at our institution were instructed when in doubt to accept every detail encountered in the OSCE as pertaining to the simulation and had encountered moulages several times before in their program prior to the OSCEs analysed here. Obviously, moulages should add to a simulation’s credibility and not reduce it. To better tackle this, we suggest scripting the SP to directly or indirectly confirm moulages as part of their condition(s), at least if they notice such hesitation by the candidates. Another way would be to implement the use of moulage even more broadly in teaching, whenever reasonable, so that the modalities of simulation are streamlined and well-known.

Another implication springs from the potential of moulages being misinterpreted as part of reality. An SP, wearing a transfer of melanoma all day during an exam, perhaps having listened to multiple examinees that the SP’s itchy skin lesion is in fact cancerous and needs urgent treatment, might require special attention during debriefing, perhaps more so than if the skin lesion had only been presented as a photograph and physically separate from the SP. This aspect of the use of moulages in simulations should be evaluated in relation to the suggestions that moulages might help SP engage more with their role and the simulation [18], [19]. 

The approaches and results reported here are based on three years of experience in which about 40 three-dimensional transfers were deployed in teaching and assessment. It can be assumed that people new to the process of producing moulages as described will need several attempts until they achieve acceptable outcomes. Cost-benefit considerations should not only take human and financial resources into account but also the moulages’ intended role in simulations. However, if an educator or an institution of health professions education decide to use moulages in their simulation program, then the development and production must follow a strict policy of quality assurance and collect feedback whenever possible. 

Possible next steps not only include revisiting the materials and methods used in the development of three-dimensional transfers to prevent lint on sticky edges, but also considering three-dimensional printing technique in the production process or the possibilities of augmented reality. Furthermore, how learners’ perceive the gestalt of the simulation encounter when using hyper-realistic moulages warrants additional attention. The same goes for the question of how such moulages influence SP in adopting and shedding their role. 

## 5. Conclusion

The step-by-step approach reported can be adapted and replicated by other healthcare educators to build their own three-dimensional transfers. This should widen the scope of their simulations and the simulations’ lifelikeness. At the same time we propose this level of lifelikeness needs to become the default expectation by learners in future. Our evaluation of their use in high-stakes assessments implies their usefulness and acceptance.

## Acknowledgements

The authors would like to extend their gratitude to all SP, SP trainers, and clinician experts who contributed to the development of our moulages.

## Notes

### Shared authorship

The authors Sandra Wüst and Daniel Bauer share the last authorship.

### Authors’ ORCIDs

- Daniel Bauer: [0000-0002-3337-3327]
- Beate Brem: [0000-0002-0551-9587]
- Helmut Beltraminelli: [0000-0002-8179-1793]
- Andrea Lörwald: [0000-0002-4217-8101]
- Kai Schnabel: [0000-0002-6977-2717]

### Ethics

No ethics approval was necessary under the Swiss Law on Human Research. The evaluations collecting the analysed data were routine measures of quality control. 

## Competing interests

The authors declare that they have no competing interests. 

The authors are not obliged to any of the named companies. Upon request, the Institute for Medical Education produces moulages on a not for profit basis for health professions education institutions.

## Supplementary Material

Supplementary material

## Figures and Tables

**Table 1:**
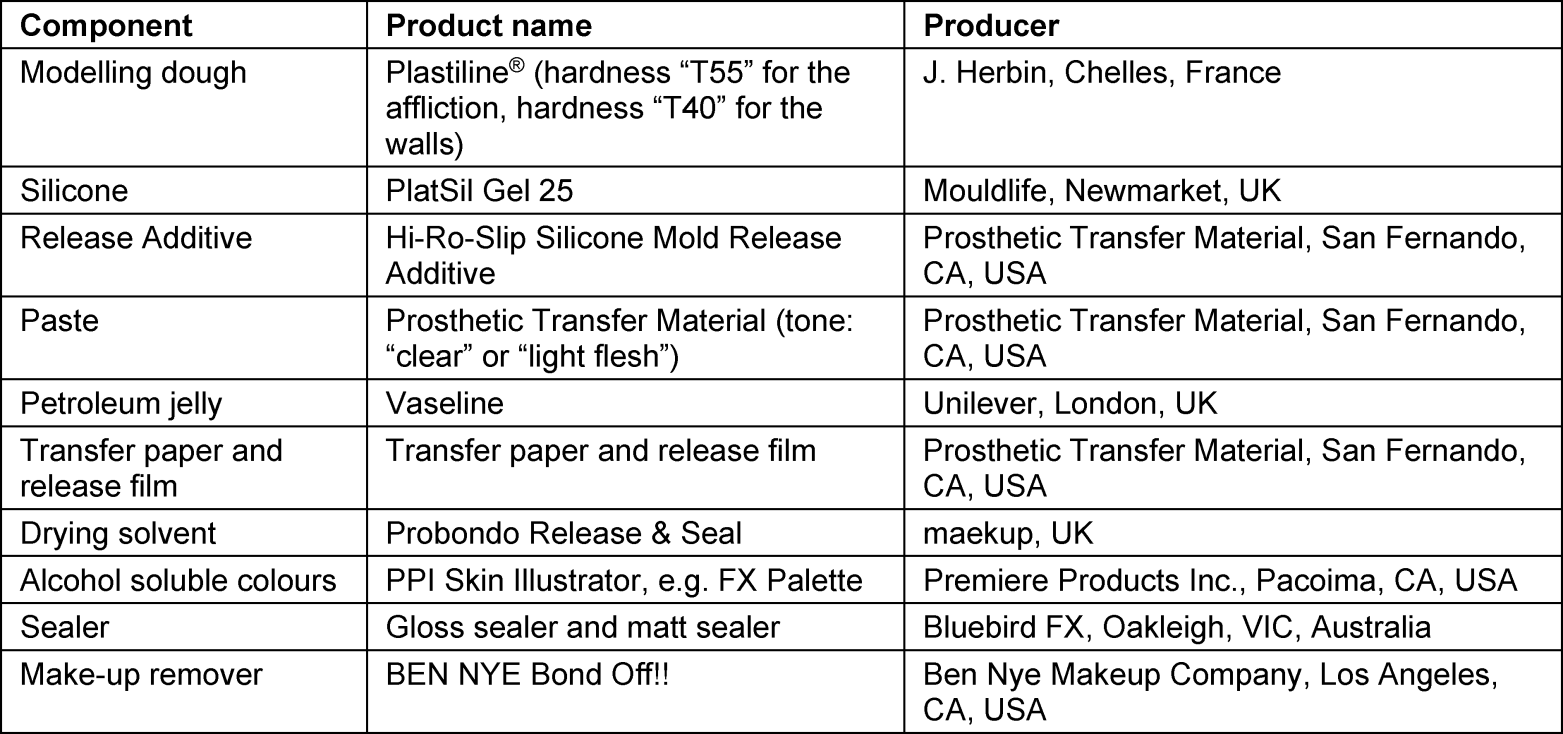
Products used, including producers

**Table 2:**
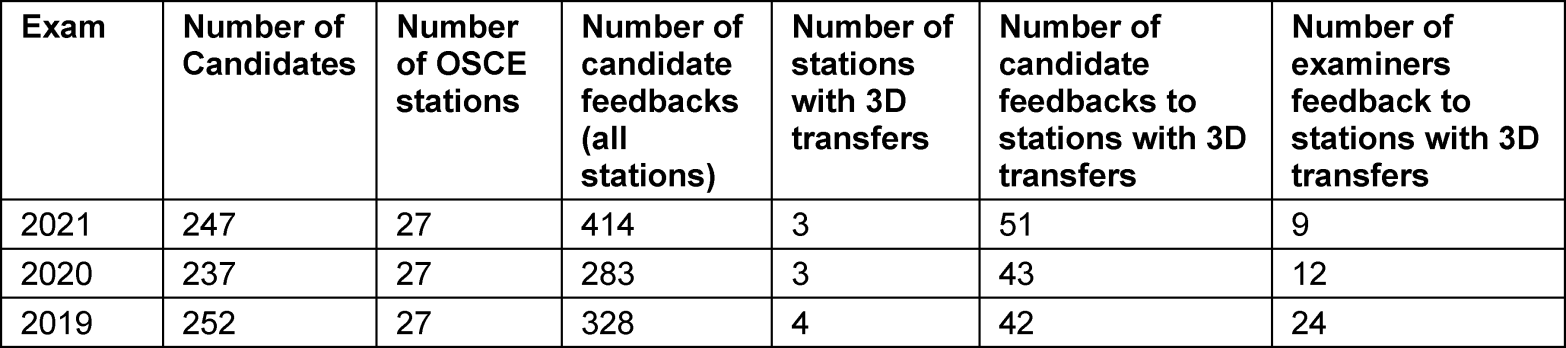
Evaluation of students’ and examiners’ feedback on three OSCEs utilizing three-dimensional transfers
