# Supplementary material for: Development and evaluation of three-dimensional transfers to depict skin conditions in simulation-based education
Source: GMS J Med Educ. 2024 Apr 15;41(2):Doc14. doi: 10.3205/zma001669 (PMC11106571; doi:10.3205/zma001669)
Supplement: Supplementary material [file JME-41-14-s-001.pdf]

## Attachment 1: Supplementary material

Attachment 1 to Schnabel KP, Lörwald AC, Beltraminelli H, Germano M, Brem BG, Wüst S, Bauer D. *Development and evaluation of 3-dimensional transfers to depict skin conditions in simulation-based education.* GMS J Med Educ. 2024;41(2):Doc14. DOI: 10.3205/zma001669

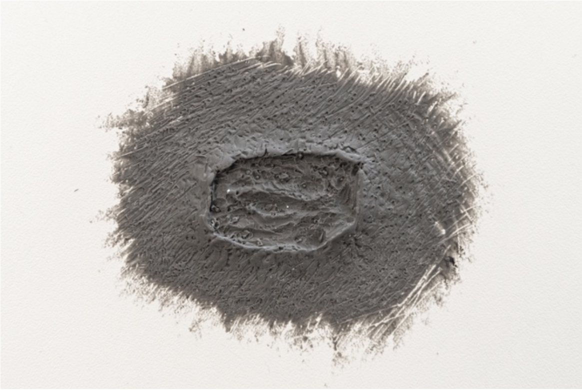

**1)** Positive model made from modelling dough.

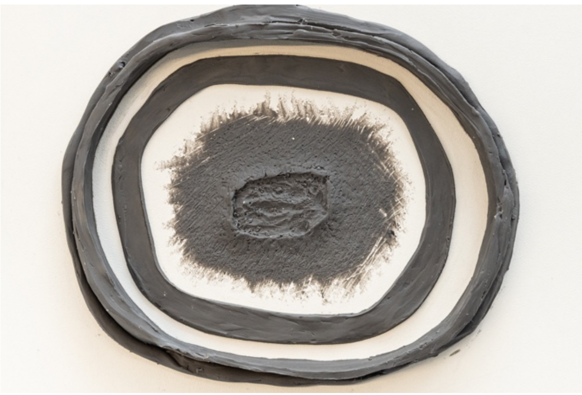

**2)** Added two walls, ready for pouring.

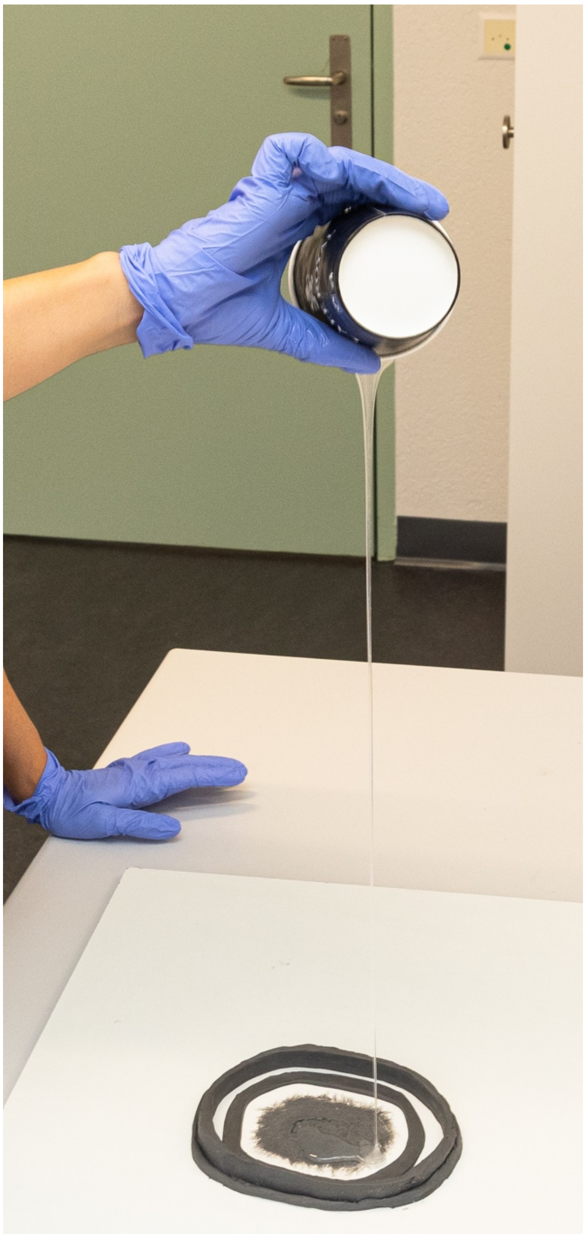

**3)** Pouring the silicone

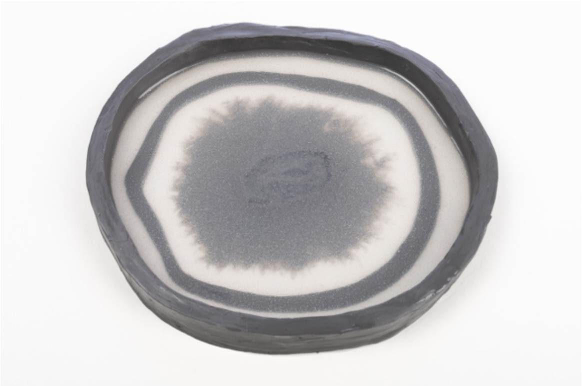

**4)** The silicone covers the inner but not the outer wall.

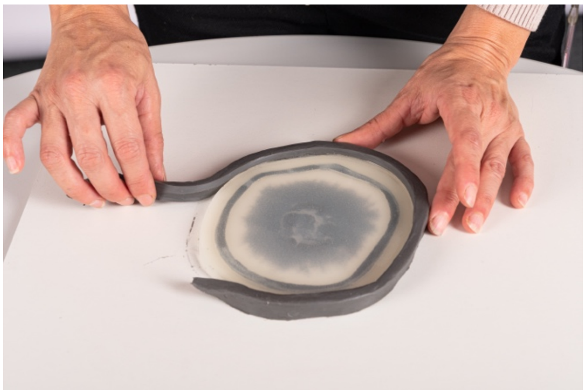

**5)** Removing the wall

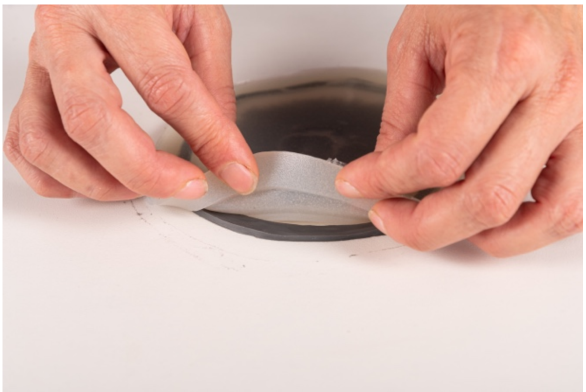

**6)** Removing the (negative) silicone cast mould.

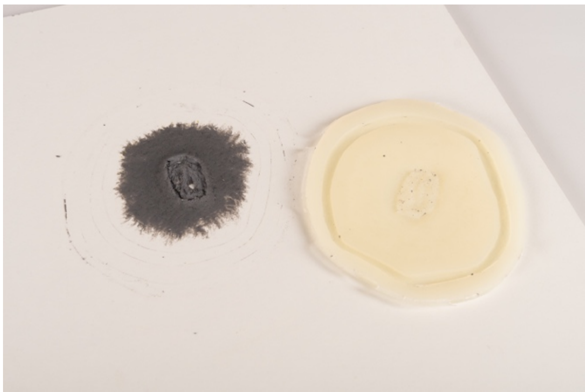

**7)** Positive and negative.

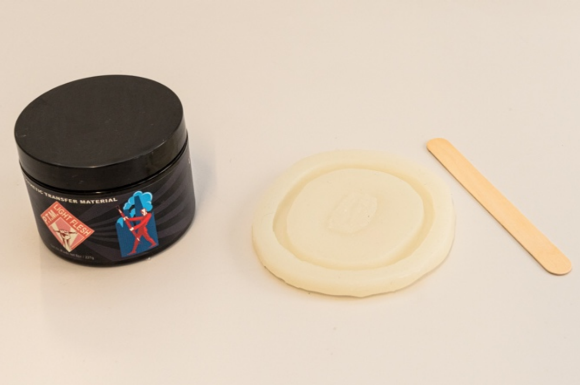

**8)** Material for the transfer.

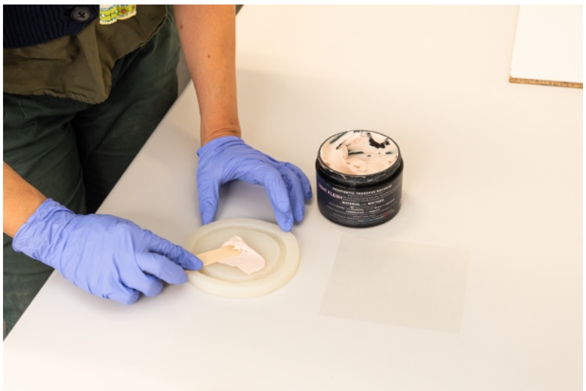

**9)** Filling in the transfer paste.

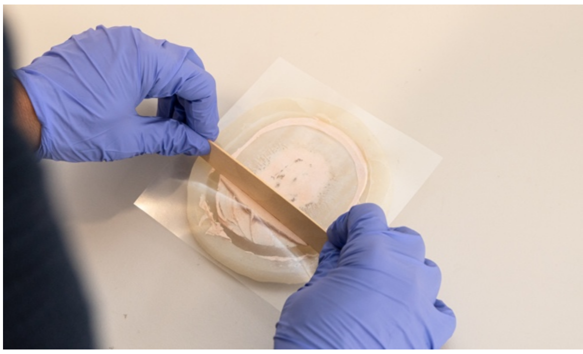

**10)** Having added the release film, the transfer paste is spread.

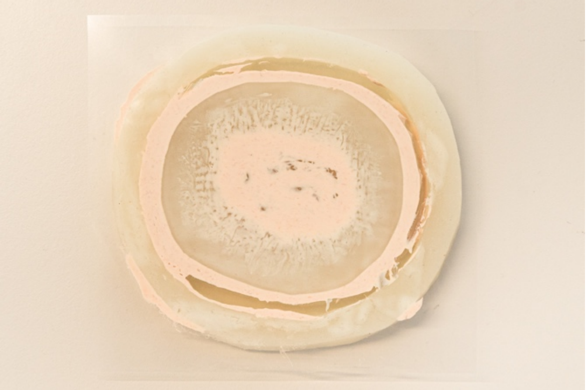

**11)** Ready for deep freeze.

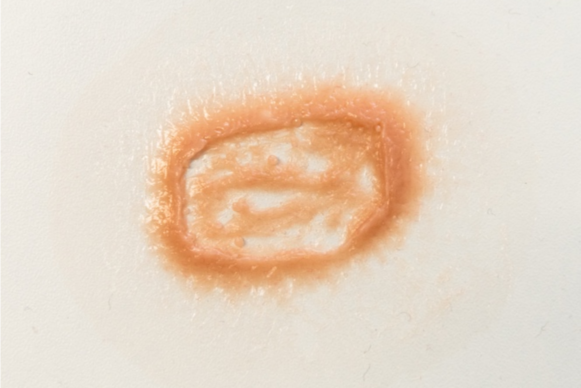

**12)** Transfer has been removed, ready for colouring

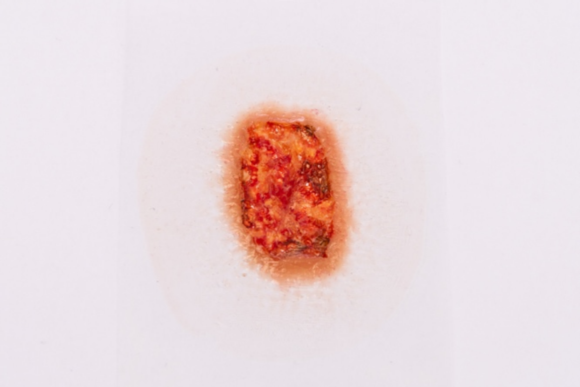

**13)** Coloured transfer.

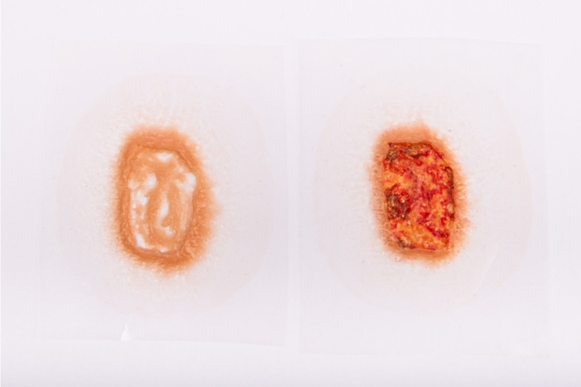

**14)** Raw and coloured transfer.

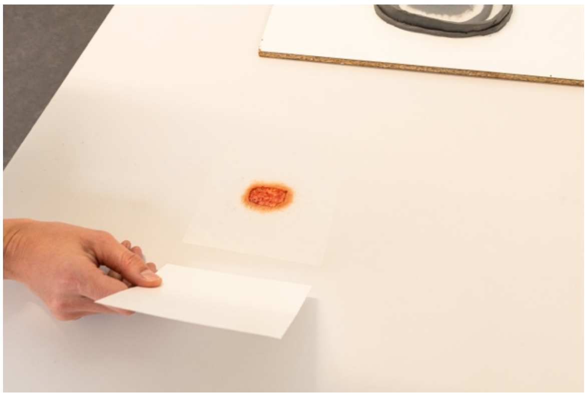

**15)** Preparing the transfer paper.

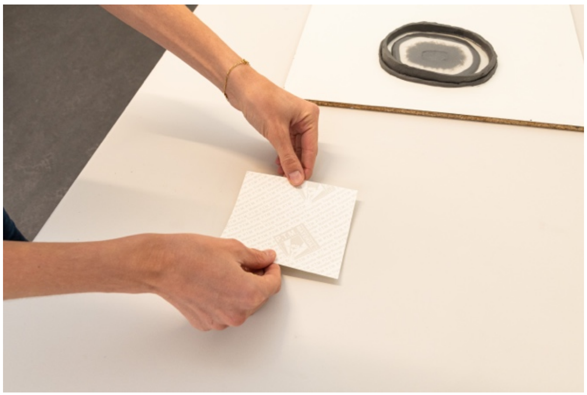

**16)** Placing the transfer paper on the transfer.

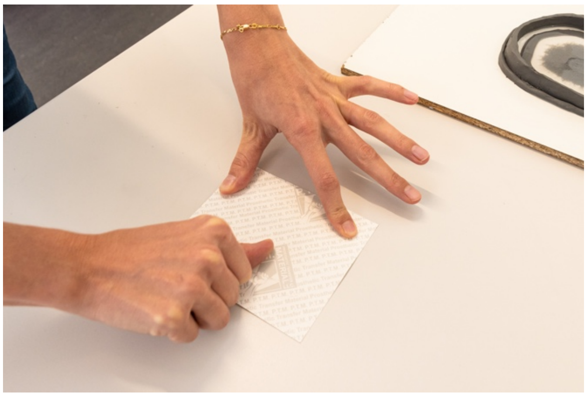

**17)** Apply pressure from the back.

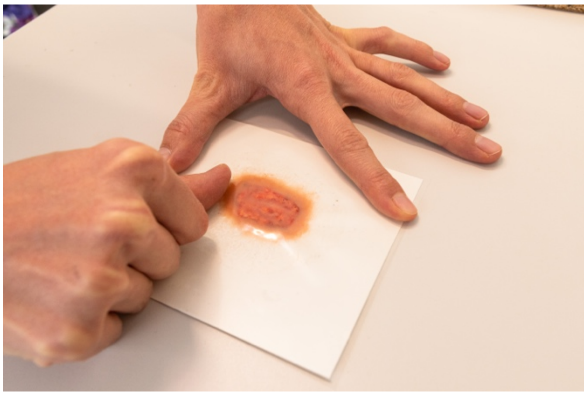

**18)** Apply pressure from the front.

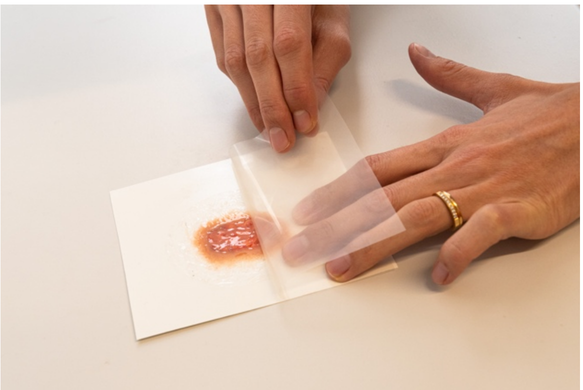

**19)** Remove release film before applying on person.

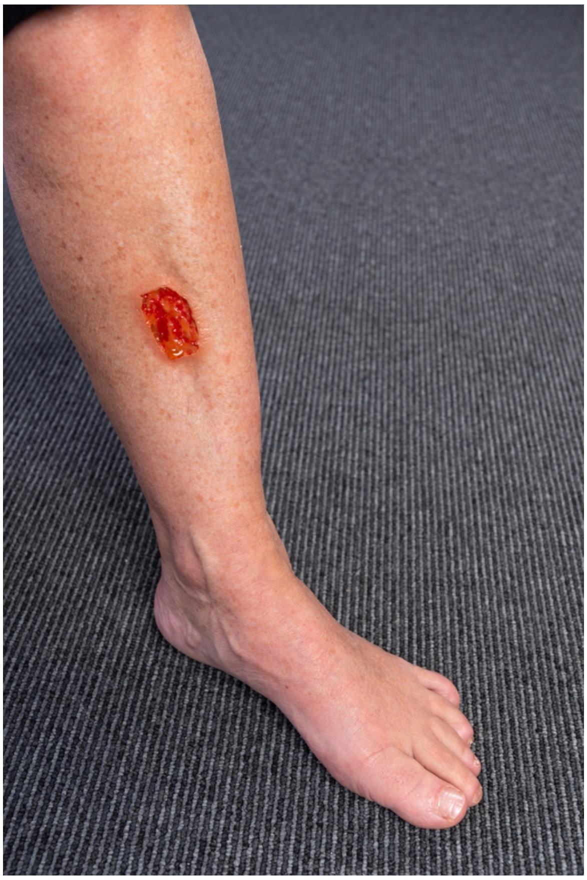

**20)** The finished transfer on the SP's leg.
